# Tone Burst Electrocochleography for the Diagnosis of Clinically Certain Meniere's Disease

**DOI:** 10.3389/fnins.2017.00301

**Published:** 2017-06-16

**Authors:** Jeremy Hornibrook

**Affiliations:** Department of Otolaryngology-Head and Neck Surgery, Christchurch Hospital, University of Canterbury and University of OtagoChristchurch, New Zealand

**Keywords:** Meniere's disease, electrocochleography, tone bursts, transtympanic EcochG, clinically certain Meniere's disease

## Abstract

The technique of transtympanic electrocochleography was initially developed as an objective hearing threshold test by Eggermont. Gibson et al. (1977) claimed that an enlarged direct current component of the action potential (AP) called the summating potential (SP) is an indication of endolymphatic hydrops, later confirmed by Coates who proposed an SP/AP ratio measure. This led to numerous publications using diagnostic ratios of 0.33–0.35. The insensitivity led to an eventual disenchantment with the test as a reliable objective test for Meniere's disease. It was further confused by audiologists employing remote canal or ear drum electrodes which give a response about one-fourth of the magnitude obtained by an electrode in contact with the cochlea. Subsequently Gibson stated that an SP/AP ratio of <0.5 is not diagnostic for hydrops. He then showed that a tone burst stimulus gave the test a significantly higher sensitivity and specificity, which has been supported by others. On MRI inner ear imaging with gadolinium hydrops can be seen, but the quality of images and what is seen may vary according to brand of scanner, settings, mode of gadolinium administration, and the possibility that gadolinium entry may favor the vestibule. Transtympanic tone burst electrocochleography is to date the simplest, cheapest and most sensitive technique for detecting cochlear endolymphatic hydrops to confirm a diagnosis of Meniere's disease.

## Introduction

Electrocochleography (EcochG) is a method of directly recording electrical activity of the cochlea and the acoustic nerve in response to acoustic stimulation. The three components measurable are the cochlear microphonic (CM), the action potential (AP), and the summating potential (SP). In contrast to the earliest studies, new computer averaging techniques have enabled routine testing of these components in humans.

This review will briefly summarize the useful components of the EcochG used in the diagnosis of Meniere's disease. The effects of electrode placement on the size of the AP and SP and the merits of tone burst stimuli will be discussed. New international criteria for the symptomatic diagnosis of Meniere's disease make no allowance for any diagnostic test for a disorder which always begins in the cochlea, even though objective testing can confirm or exclude it.

## Cochlear microphonic

The CM, originally called the cochlear potential, was recorded in cats by Wever and Bray (1930). It is thought to be the summed microphonic from many hair cells recorded by a distant electrode. The lower the frequency of stimulation the larger the number of hair cells which will produce CMs in the same phase and the larger the CM will be. Although the CM has a number of new applications in auditory testing, its routine use is somewhat limited by the reduction in signal-to-noise ratio that occurs with a remote electrode.

## Action potential

The response from the acoustic nerve is the AP and was first demonstrated in the cochlear nerve and brainstem of cats by Saul and Davis (1932). Because of the concern that direct recording from the cochlea in individuals with normal hearing was dangerous, Ruben et al. (1961) measured APs in patients with hearing losses by a silver ball electrode placed in the round window niche after a typmanotomy and achieved the first intraoperative demonstration of hearing improvement during stapedectomy. In what was the first use of a remote electrode, Yoshie et al. (Yoshie, 1968) measured APs in normal hearing humans with a hypodermic needle shielded with a polyethylene tube inserted into the anesthetised posterior ear canal skin, about 5 mm from the annulus. In the same year Portmann (Portmann et al., 1967) demonstrated that it was safe to record from the round window niche with an electrode passed through the eardrum. In the USA fears of safety and litigation over transtympanic electrodes persist to this day.

A 100 ms click stimulus stimulates the whole basilar membrane. Frequency selective masking experiments suggest that the major contribution from a click is from the basal turn of the cochlea (Teas et al., 1962) from 10 to 4 kHz as the traveling wave is progressively damped as it travels toward the apex (Zwicker and Fast, 1972). Also the velocity of the traveling wave along the basilar membrane slows as it approaches the apex of the cochlea, resulting in a decrease in hair cells firing per unit time (Zerlin, 1969). This limitation is being addressed by the study of an alternative “Chirp” stimulus which has more low frequency energy occurring earlier in the stimulus (Chertoff et al., 2010).

The initial application of AP recordings was the objective determination of hearing thresholds. As the signal is generated so close to the recording electrode, masking of the opposite ear is not necessary.

## Summating potential

The SP is a direct current component of the AP, described independently in guinea pigs by Davis et al. (1950) who assumed it was a post-synaptic response. von Bekesy (1952) considered it to be a shift of the CM. The CM was thought to be derived from the outer hair cells and the SP from the inner hair cells. However, it is present in pigeon ears which lack inner hair cells (Stopp and Whitfeild, 1964). The SP is now assumed to be a result of cochlear microphonic distortions (Dallos et al., 1972).

The maximum CM is recorded closest to maximum hair cell displacement, whereas the SP is maximum at a point where the summed effect from a large area of basilar membrane can be recorded. In endolymphatic hydrops the downward vibration of the basilar membrane is limited as it is being stretched, so the normal up-going asymmetry is enhanced, leading to a SP of increased amplitude (Gibson, 1978).

The SP became to be of interest as an indicator of endolymphatic hydrops, and therefore in the objective diagnosis of Meniere's disease.

## Summating potential in meniere's disease

Schmidt et al. (1974) noted that the SP in Meniere's disease from tone bursts is about five times larger than in patients with high frequency hearing loss. Eggermont (1979) found that short 4 kHz 4 ms tone pips elicit a small AP which limits their use for diagnosing Meniere's disease compared with a click stimulus (Gibson, 1978).

Gibson et al. (1977), using transtympanic EcochG with clicks, found a large DC potential causing a widening of SP/AP waveform that might be a useful indicator of Meniere's disease. There was a high correlation with the symptomatic likelihood of Meniere's disease. Moffat et al. (1978) achieved a decrease in the negative SP in 11/13 patients after oral glycerol dehydration, with no significant change in the pure tone audiogram or speech discrimination. This was suggested as being a useful indicator of prognosis in endolymphatic sac surgery.

Coats (1981a) found that Meniere's ears had a larger SP/AP ratio compared with non-Meniere's ears, when recorded using a canal electrode and a click stimulus. There was also a correlation between a large SP/AP ratio in ears with reduced caloric responses in comparison with a small SP/AP ratio in ears with normal caloric responses (Coats, 1981b).

A major issue of contention for the EcochG has been the magnitude and quality of responses depending on the type and placement of the active electrode.

## Electrode placement

The majority of publications on click stimulus in Meniere's ears have been by audiologists using distant electrodes which, because of their distance, require more signal averaging to cancel out random noise, and produce far smaller responses.

Ferraro et al. (1986) compared the responses and comfort of three ear canal electrodes.

Of the three there was no difference in comfort. A disposable soft insulated ear canal foam plug electrode design with a central sound-conducting tube was the easiest to place and gave the best responses. Sohmer and Feinmesser (1967) recorded the AP in cats with silver ball electrode in the round window niche and the ear drum and from a subdermal needle and a clip on the ear lobe. He found that the AP recorded from the round window niche was 10–25 times larger than the AP recorded from the other three sites.

Roland et al. (1995) compared responses from a transtympanic electrode (TT) with an ear canal (EAC) electrode in 19 healthy volunteers. The click responses from a TT electrode were seven times the magnitude as those from a EAC electrode. In a further study 50 ear canal EcochG tracings interpreted by 10 different audiologists revealed statistically significant inter-interpreter differences between no response and very difficult to read SP/AP ratios (Roland and Roth, 1997). He emphasized the implications for diagnosis and its reliability in investigational studies.

## Click SP/AP studies in meniere's disease

Gibson et al. (1983) performed click stimulus EcochG in 44 Meniere's ears and in 32 normal ears and 40 ears with sensorineural hearing loss. A SP/AP ratio of 0.30 clearly separated them, providing the loss exceeded an average of 40 dBHL.

The click SP/AP ratio as a diagnostic test for Meniere's disease became of world-wide interest and the basis of numerous publications, some of which are listed in Table 1. The highest sensitivity of 85% was achieved by Camilleri and Howarth (2001) with an SP/AP ratio of 0.33. In contrast Gibson et al. (Gibson, 2005) reported a 40% sensitivity with an SP/AP ratio of 0.47. The explanation for this will follow. In addition to a click SP/AP ratio Ferraro and Tibbils (1999) recorded the AP using an ET electrode. He advocated the addition of an SP/AP area ratio (Ferraro and Tibbils, 1999) to improve the sensitivity and specificity to 92 and 84%, respectively (Al-momani et al., 2009). However, Marcio et al. (transtympanic) (Marcio et al., 2006) and Ikino et al. (transtympanic) (Ikino and de Almeida, 2006) could not confirm it.

**Table 1:** SP/AP ratio criteria from extratympanic (ET) and transtympanic (TT) EcochG studies with a click stimulus.

**Authors**	**Electrode**	**SP/AP criterion**	**Sensitivity (%)**	**Specificity (%)**
Mori et al., 1987	ET	0.44	68	
Aso, 1991	TT	0.37	58	
Pou et al., 1996	ET	0.35	57	94
Filipo et al., 1997	TT	0.43	64	
		0.50	47	
Sass, 1998	TT	0.41	62	95
Ferraro and Tibbils, 1999	ET	0.41	60	
Camilleri and Howarth, 2001	TT	0.33	85	
Chung et al., 2004	ET	0.34	71	96
Gibson, 2005	TT	0.47	40	97
Marcio et al., 2006	TT	0.37	52	
Takeda and Kakigi, 2010	ET	0.40	56	
Claes et al., 2011	TT	0.35	56	

A significant advance in the EcochG sensitivity for diagnosing definite Meniere's disease has come from the use of tone burst stimuli.

## Tone burst studies in meniere's disease

In 1986 Dauman et al. (Dauman et al., 1986, 1998) measured the effect of glycerol on ears tested transtympanically with free field tone bursts of octave frequencies between 1 and 8 kZ at 90 dB HL, which produced a prolonged SP whose magnitude was measured in microvolts from its midpoint to the baseline. Long tone bursts in patients with Meniere's disease showed significantly larger SPs than in control subjects, with most Meniere's ears having an SP decrease observed after dehydration.

**Figure 1:**
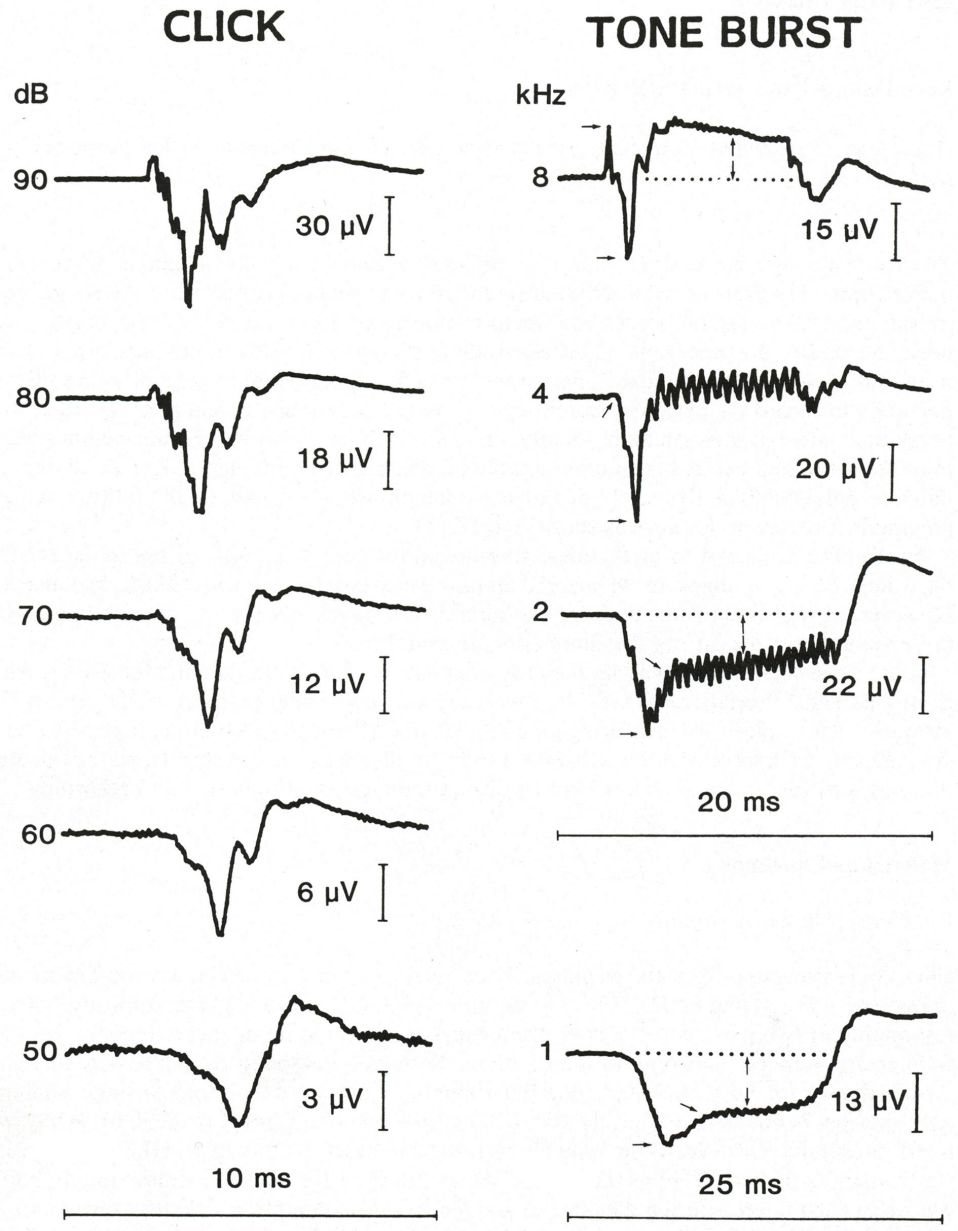
Transtympanic SP responses to a 90 dB click and to 10 ms 1, 2, 4, and 8 kHz tone bursts with 8 kHz showing a reversed polarity. The magnitude is measured in microvolts from the midpoint of the prolonged SP to the baseline (Dauman and Aran, 1991). Reproduced from Dauman and Aran (1991).

**Figure 2:**
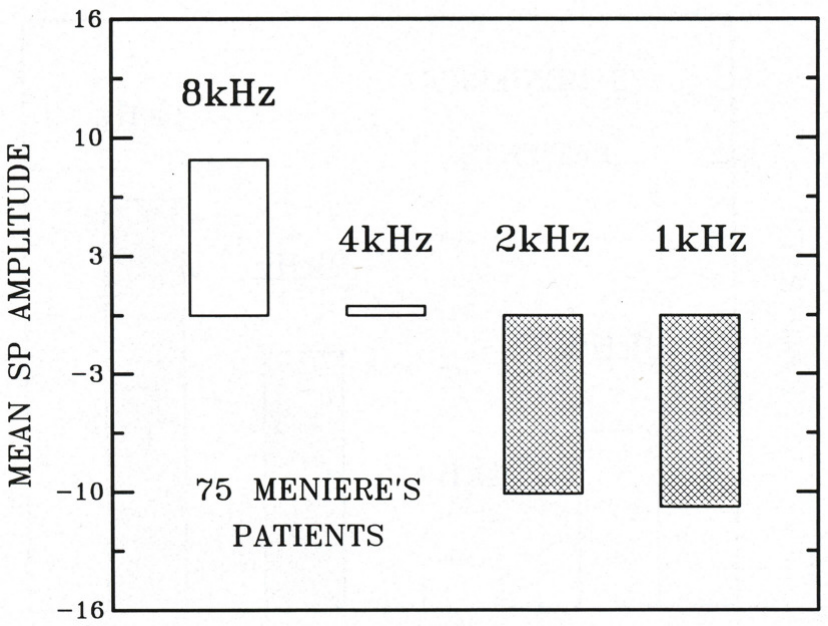
The mean tone burst SP amplitudes (in mV) in 75 Meniere's patients at 1, 2, 4, and 8 kHz, respectively (Dauman and Aran, 1991). Reproduced from Dauman and Aran (1991).

In 1990, at the Third International Symposium and Workshops on Surgery of the Inner Ear, Dauman and Aran (1991) expanded their experience, comparing clicks vs. 10 ms tone bursts. The responses to 1, 2, 4, and 8 Kz TBs are shown in Figure 1, with 8 kHz usually being positive. The mean amplitudes for those frequencies are shown in Figure 2, showing 1 and 2 kHz are the most sensitive for indicating hydrops. Gibson (1991) compared clicks with 1 kHz 12 ms tone bursts in 42 Meniere's ears and 48 normal sensorineural loss ears, with the symptomatic likelihood of Meniere's disease. At 90 dB HL a 1 kHz tone burst more negative than 3 mV separated the Meniere's ears very precisely from the normal and sensorineural ears. The false negatives for tone bursts were half those for clicks.

At The First International Conference on EcochG, Otoacoustic Emissions, and Intraoperative Monitoring Gibson (1993) expanded the comparison of clicks vs. tone bursts (12 ms) for the diagnosis of endolymphatic hydrops in 1,101 ears by transtympanic EcochG. The 0.5, 1, 2, 4, and 8 kHz tone burst diagnostic criteria are presented in Table 2.

**Table 2:** Diagnostic level for tone bursts to diagnose hydrops (Gibson, 1993).

**Hz of SP hearing level**	**dB HL**	**Diagnostic criterion (mV)**
**MORE NEGATIVE THAN**		
500 HZ (85 dB HL)	under 25	−2
	20–35	−2
	40–55	−2
	60–75	−1
1 kHz (100 dB HL)	under 25	−6
	20–25	−6
	40–55	−6
	60–75	−3
2 kHz (100 dB HL)	under 25	−9
	20–35	−7
	40–55	−5
	60–75	−5
4 kHz (100dB HL)	under 25	−9
	20–35	−5
	40–55	−5
	60–75	−5
**MORE POSITIVE THAN**		
8 kHz (100 dB HL)	under 25	+6
	20–35	+6
	40–55	+6
	60–75	+6

*The diagnostic level was chosen as the nearest whole figure to the level which provides a false rate of 5%*.

Conlon and Gibson (2000) confirmed the superiority of tone bursts over clicks and with a 1 kHz tone burst found hydrops in 10% of contralateral ears in Meniere's patients (Conlon and Gibson, 1999). Claes et al. (2011) used a transtympanic technique with 100 dBHL tone bursts. He achieved a 91% sensitivity for implying hydrops in 91% of ears with an AAO-HNS definite diagnosis of Meniere's disease when the SP amplitude was more negative than −3 mV for 1 kHz or more negative than 2 mV in at least three tone burst frequencies.

Ferraro (Ferraro et al., 1994) found tone burst SPs measured with an extratympanic electrode were four times smaller compared with a transtympanic electrode. Bohlen et al. (1991) measured click and tone burst responses sequentially with an extratympanic and transtympanic electrode. In 90% of patients TT EcochG was equal to or more comfortable than for an ET electrode. Tone bursts with an ET electrode gave no response or were unreliably small.

In most EcochG studies on Meniere's ears the control ears have been Meniere's opposite ears or ears with normal hearing or ears with sensorineural hearing loss. To provide purer controls Gibson (2009) compared click and tone burst responses in 2,717 patients from Meniere's ears *with ears with equivalent hearing*. For a click SP/AP response there was no statistical difference between Meniere's ears and non-Meniere's ears. In a further analysis (Iseli and Gibson, 2010) a click stimulus had diagnostic sensitivity of 35% and specificity of 91% for an SP/AP ratio of not <0.47, compared with and 95% sensitivity and 79% sensitivity for combination of 1 kHz tone burst thresholds and a tone burst SP/AP ratio.

Despite significant advances in the sensitivity of electrophysiological testing official diagnostic classifications for Meniere's disease remain symptom-based.

## Current diagnosis of meniere's disease

Since Prosper Meniere's first descriptions of the disorder in 1861 there was no recognized symptomatic classification until 1972 (Barber et al., 1972). The Equilibrium Committee of the American Academy of Otolaryngology-Head and Neck Surgery (AAO-HNS) has produced three diagnostic definitions, the most recent one in 1995 (Monsell et al., 1995) used internationally until 2015 (Table 3). The four categories were possible, probable, definite, and certain. AAO-HNS definite has been a universal diagnostic criterion for numerous clinical studies. The definition of certain was histopathological confirmation from a post-mortem. The AAO-HNS has been and remains skeptical as to the reliability of any objective tests.

**Table 3:** AAO-HNS Committee on Hearing and Equilibrium 1995 diagnostic criteria for Meniere's disease (Monsell et al., 1995).

**CERTAIN MENIERE'S DISEASE**
Definite Meniere's disease, plus histopathologic confirmation
**DEFINITE MENIERE'S DISEASE**
Two or more definitive spontaneous episodes of vertigo of 20 min or longer
Audiometrically documented hearing loss on at last one occasion
Tinnitus or aural fullness in the treated ear
Other causes excluded
**PROBABLE MENIERE'S DISEASE**
One definite episode of vertigo
Audiometrically documented hearing loss on at least one occasion
Tinnitus or aural fullness in the treated ear
Other causes excluded
**POSSIBLE MENIERE'S DISEASE**
Episodic vertigo of the Meniere type without documented hearing loss, or
Sensorineural hearing loss, fluctuating, or fixed, with disequilibrium but without definitive episodes
Other causes excluded

The Barany Society, an international vestibular disorders society based in Sweden, has embarked on a project to achieve worldwide agreement on precise definitions of vestibular symptoms and the symptomatic diagnosis of common vestibular disorders. To conform to The International Classification of Diseases the vestibular diagnoses are limited to probable and definite. For definite Meniere's disease the new symptomatic criteria (Lopez-Escamez et al., 2015) are similar and a logical improvement on the AAO-HNS 1995 criteria (Table 4). Possible and certain no longer exist.

**Table 4:** The 2015 Barany Society diagnostic criteria for Meniere's disease (Lopez-Escamez et al., 2015).

**DEFINITE MENIERE'S DISEASE**
A. Two or more spontaneous episodes of vertigo, each lasting 20 min to 12 h
B. Audiometrically low-to-medium-frequency sensorinerual hearing loss in one ear, defining the affected ear on at least one occasion, during or after one of the episodes of vertigo
C. Fluctuating aural symptoms (hearing, tinnitus, or fullness) in the affected ear
D. Not better accounted for by another vestibular diagnosis
**PROBABLE MENIERE'S DISEASE**
A. Two or more episodes of vertigo or dizziness, each lasting 20 min to 24 h
B. Fluctuating aural symptoms (hearing, tinnitus, or fullness) in the affected ear
C. Not better accounted for by another vestibular diagnosis

## Opinion on the validity of EcochG for the diagnosis of meniere's disease

Nguyen et al. (2010) conducted a survey among members of the American Otological Society and the American Neurotology Society as to their opinions on the usefulness of EcochG for diagnosing Meniere's disease. Approximately 70% employed an extratympanic electrode and 30% a transtympanic electrode. Eighty-three percent said they would discount a result that was contradictory to their clinical impression, with 57% preferring an ENG caloric test and VEMPs for 27%. Only 45% used EcochG. The overall conclusion was that EcochG is perceived to have low clinical use and reliability, and among those who use it there is little consensus on technique and stimulus modality.

Kim et al. (2005) conducted a click EcochG study with an extratympanic electrode and an SP/AP diagnostic ratio of >0.4 on 97 patients with suspected Meniere's disease. Of 60 patients with an AAO-HNS symptomatic diagnosis of Meniere's disease 67% with a definite diagnosis and 53% with a less-than-definite diagnosis had a positive test. They concluded that, because of its lack of sensitivity, EcochG should not play a decisive role in determining the presence or absence of Meniere's disease.

## Vestibular meniere's disease

The term vestibular Meniere's disease is sometimes used (Paparella, 1984a,b; Paparella and Mancini, 1985). It originated in the earliest iteration of the AAO-HNS diagnostic criteria (Barber et al., 1972), separating cochlear and vestibular forms, but abandoned in the 1995 criteria. Currently it has no official basis.

Dornhoffer and Arenberg (1993) studied 15 patients with recurrent vertigo attacks without fluctuating hearing they called vestibular Meniere's disease (or possible on the 1995 AAO-HNS criteria). On transtympanic tone burst EcochG at 1 and 2 kHz by their own criteria 6 were positive for hydrops, supporting a diagnosis of Meniere's disease.

With the abolition of the AAO-HNS certain Meniere's disease category (a post-mortem now rarely achievable) there is a need for alternative diagnostic certainty, particularly for investigational studies, and to unequivocally distinguish Meniere's disease from other causes of recurrent vertigo attacks.

## Clinically certain meniere's disease

The term clinically certain Meniere's disease can be defined as a diagnosis based on the 1995 AAO-HNS symptomatic criteria (Monsell et al., 1995) (or now probable and definite on the international Barany Society criteria (Lopez-Escamez et al., 2015) plus transtympanic electrocochleographic confirmation of endolymphatic hydrops, based on the most sensitive criteria for tone bursts and clicks.

**Figure 3:**
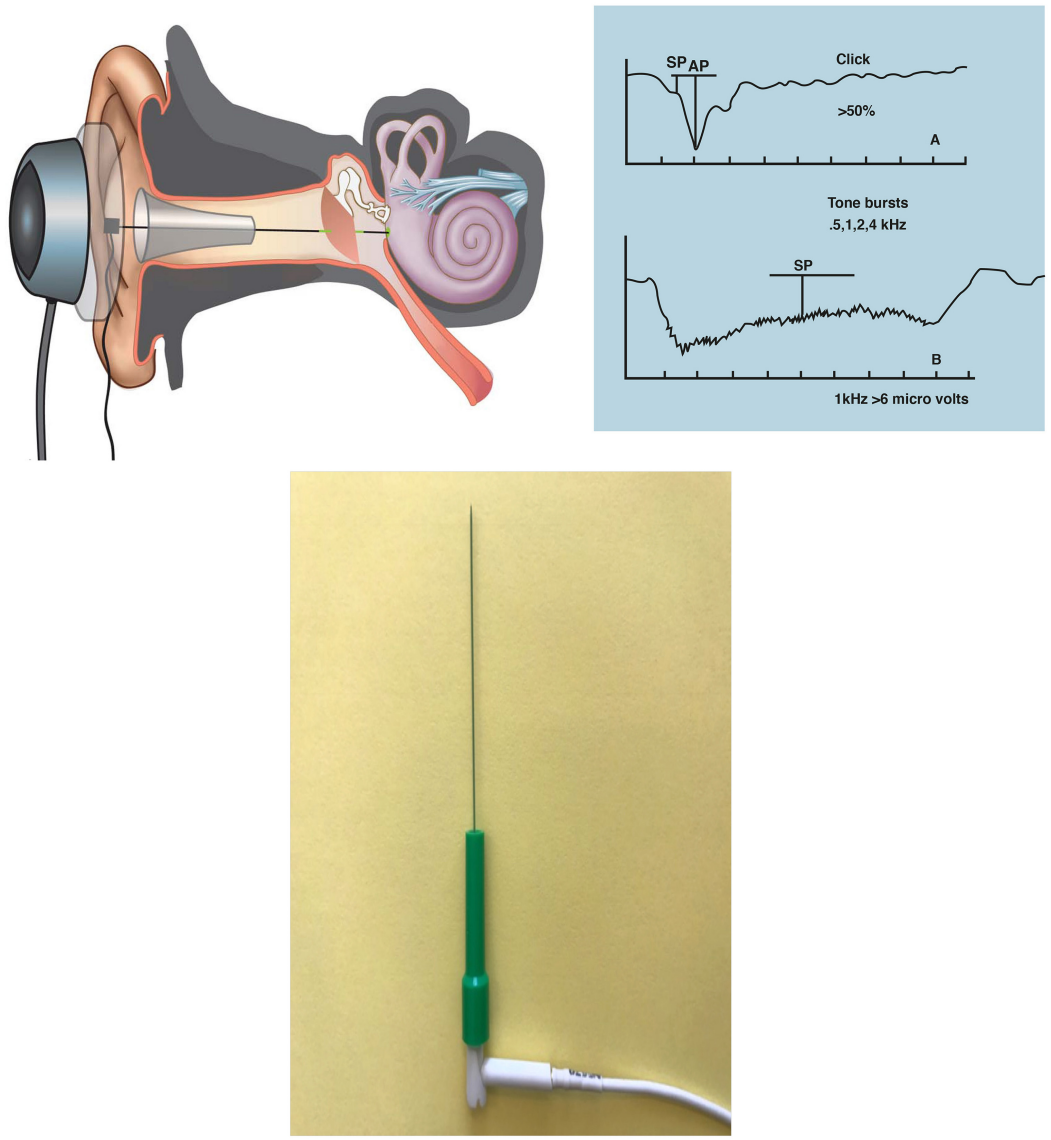
Transtympanic EcochG for three studies on “clinically certain” Meniere's disease (Hornibrook et al., 2010b, 2011; Johnson et al., 2016). Local anesthesia is a tiny drop of phenol placed at 9 o'clock or 3 o'clock on the ear drum. An insulated needle is passed through the drum with its tip lying in the round window niche and secured by elastic bands to a circular bracket over the ear over which a headphone is placed. Stimuli were from an Amplaid mk15 diagnostic system. Clicks: 10 ms broadband alternating polarity clicks of 100 ms duration at 95 dB HL at repetition rate of 11.3/s with 100–200 repetitions. Tone bursts: 1 and 2 kHz 100 dB HL tone bursts with a 1 ms linear rise/fall and 14 ms plateau at a repetition rate of 30.1/s. The diagnostic criterion for clicks was an SP/AP ratio of >0.5, and for tone bursts criteria for 1 and 2 kHz as in Table 2 (Gibson, 1991). From Hornibrook et al. (2012). **Inset:** TECA disposable monopolar needle 37 mm x 28 G (Natus Manufacturing Ltd., Gort, Galway, Ireland).

Based on this definition Hornibrook (Hornibrook et al., 2010b, 2011; Johnson et al., 2016) and colleagues have conducted three studies on definite Meniere's disease patients in whom there was clinical certainty of the diagnosis. Objective proof of hydrops was established by transtympanic EcochG with tone bursts and clicks. The technique and settings are illustrated in Figure 3. The diagnostic tone burst criteria were at 1 and/or 2 kHz (Table 2; Gibson, 1993) and/or a click SP/AP ratio of >0.5.

Since the discovery that an abnormally low threshold cervical vestibular evoked potential (cVEMP) could confirm a diagnosis of superior canal dehiscence syndrome other diagnostic applications for VEMPs have been sought, including for Meniere's disease with numerous publications employing cVEMPS and ocular VEMPs (oVEMPs) to diagnose hydrops in the vestibule. These have produced conflicting interpretations as to the diagnostic sensitivity. In 18 patients with a clinically certain diagnosis in one ear, Johnson et al. (2016) measured cVEMP and oVEMP amplitude, latency and threshold in the Meniere's ear and their opposite ears and in the ears of 22 normal control ears. The overlap of results from the Meniere's patients compared with normal controls was such that VEMP abnormalities appear limited as a sole diagnostic test for Meniere's disease. As endolymphatic hydrops in Meniere's disease always starts in the cochlea (Pender, 2014) it would seem logical to employ the most sensitive test which confirms cochlear hydrops.

Confirmation of visible inner ear hydrops on MRI scanning with intratympanic gadolinium (Nakashima et al., 2009) has led to numerous MRI inner ear studies in the hope that a visible diagnosis of hydrops would be the standard by which other tests might be compared (Hornibrook et al., 2010a).

Hornibrook et al. (2015) compared the sensitivity of intratympanic gadolinium MRI with tone burst EcochG for diagnosing hydrops in 57 ears with AAO-HNS possible, probable, or definite Meniere's disease. In 30 patients with definite Meniere's disease the tone burst EcochG was positive in 83%, the click in 30%, and gadolinium MRI in 47%. Although adequate imaging was achieved in 90% of scans, with tone burst EcochG was a more sensitive test for definite Meniere's disease and therefore for cochlear hydrops. Tone burst EcochG was also more sensitive than MRI for probable and possible Meniere's disease and in some cases, with visible vestibular hydrops, more sensitive for confirming cochlear hydrops. Ziylan et al. (2016) reviewed and compared this study with three other MRI/click-only EcochG studies with a low SP/AP diagnostic ratio of >0.33 which will have enhanced its apparent sensitivity. They concluded that there is a relative low sensitivity and predictive value for click stimulus EcochG compared with gadolinium inner ear MRI for detecting hydrops in Meniere's disease. Images and conclusions from MRI inner ear imaging appear confounded by variables such as scanner brand, head coil specifications, and the possibility that gadolinium entry may be variable and favor the vestibule (Hornibrook et al., 2016).

## Summary and conclusion

The initial promise of a click response SP/AP ratio as a sensitive test for endolymphatic hydrops has not been realized (Hornibrook et al., 2016). Although it can be measured by a ET electrode the responses are at least one quarter the magnitude of those obtained by a TT electrode.

ET electrodes are significantly inferior for measuring tone burst responses. Until the signal-to-noise ratio problem of ET electrodes is solved, TT recordings are of greater magnitude and accuracy.

An analysis of 128 Meniere's disease studies (Thorpe et al., 2003) found that the AAO-HNS 1995 definitions were misapplied in 50% of cases, implying that symptom-only criteria are unreliable and can result in underdiagnosis and overdiagnosis. Reliance on a symptom-only diagnosis, based on a pure tone audiogram, has the jeopardy that studies are likely to include patients who do not have the disorder, and to exclude some who do.

Of all investigative tests transtympanic tone burst EcochG remains the simplest, and most sensitive test to diagnose *cochlear* hydrops to confirm a diagnosis of Meniere's disease. There is agreement that a response of not < −3 mV is diagnostic for endolymphatic hydrops (Dauman and Aran, 1991; Gibson, 1991, 2005, 2009; Conlon and Gibson, 2000; Claes et al., 2011). Clear, reliable tone burst responses can only be achieved at 100 dbnHL, which cannot be achieved by newer model audiology evoked response systems.

As was once for electrocardiography there is an urgent need for universal agreement on equipment specifications (Hohmann et al., 1991; Arenberg et al., 1993; Wuyts et al., 1997), which for the EcochG should produce 100 dBnHL tone bursts.

## Author contributions

The author confirms being the sole contributor of this work and approved it for publication.

### Conflict of interest statement

The author declares that the research was conducted in the absence of any commercial or financial relationships that could be construed as a potential conflict of interest.
